# A Functional Study of the Apoptosis *Caspase* Gene Family in the Sex Differentiation of Chinese Tongue Sole (*Cynoglossus semilaevis*)

**DOI:** 10.3390/ijms27041864

**Published:** 2026-02-15

**Authors:** Lijun Wang, Haipeng Yan, Xuexue Sun, Mingyue He, Zhen Meng, Xihong Li, Na Wang, Zhongdian Dong, Wenteng Xu

**Affiliations:** 1 Guangdong Provincial Key Laboratory of Aquatic Animal Disease Control and Healthy Culture, College of Fisheries, Guangdong Ocean University, Zhanjiang 524088, China; wnglikun@163.com (L.W.); yanhaipeng265438@gmail.com (H.Y.); s1506251002@163.com (X.S.); 2 State Key Laboratory of Mariculture Biobreeding and Sustainable Goods, Yellow Sea Fisheries Research Institute, Chinese Academy of Fishery Sciences, Qingdao 266071, China; h13290100983@126.com (M.H.); mengzhen@ysfri.ac.cn (Z.M.); lixh@ysfri.ac.cn (X.L.);; 3 Laboratory for Marine Fisheries Science and Food Production Processes, Qingdao Marine Science and Technology Center, Qingdao 266071, China

**Keywords:** *Cynoglossus semilaevis*, *caspase*, high-temperature stress experiments, sex differentiation

## Abstract

Chinese tongue sole (*Cynoglossus semilaevis*) is an important aquaculture species in China. Under high-temperature conditions, genetically female fish can undergo sex reversal and develop into phenotypic males (pseudomale fish). Previous studies have demonstrated that apoptosis might function in sex differentiation. Based on this, we identified and characterized the functions of six *caspase* genes (*caspase1-like*, *caspase3a*, *caspase6*, *caspase8*, *caspase8-like*, *caspase9*) in Chinese tongue sole. These six *caspase* genes were expressed in all analyzed tissues of both males and females. They were detected to be expressed in the gonads at various developmental stages, with expression levels peaking between 7 months and 2 years of age. In situ hybridization (ISH) analysis showed that the *caspase* genes were mainly localized in spermatocytes and oocytes. Promoter activity analysis indicated that with the exception of *caspase3a*, the remaining five *caspase* genes exhibited promoter activity and were regulated by transcription factors, including *sp1* and *gata4*. High-temperature stimulation can significantly affect the expression of *caspases* in the gonads of both male and female fish, with female fish showing a more pronounced response. An siRNA-mediated knockdown experiment revealed that following *caspase* knockdown, the expression of sex differentiation-related genes, heat shock transcription factors (*hsf*), and heat shock proteins (*hsp*) in Chinese tongue sole was significantly altered. Based on these findings, we speculate that *caspases* play an important role in the sex differentiation process by responding to temperature stimuli.

## 1. Introduction

Chinese tongue sole belongs to the class *Osteichthyes*, order *Pleuronectiformes*, and family *Cynoglossidae*. It is a common large-sized warm-temperate benthic fish in China’s coastal waters. This species is highly valued in aquaculture for its superior nutritional profile and meat quality [1]. It exhibits significant sexual dimorphism, with adult females reaching two to four times the body size of males [2]. Cytogenetic karyotype analysis has revealed that the diploid chromosome number of this species is 2n = 42. The sex determination mechanism of Chinese tongue sole is the ZW/ZZ genetic system. Specifically, females possess a ZW chromosomal karyotype, whereas males have a ZZ karyotype. In addition, Chinese tongue sole exhibits a natural sex reversal phenomenon, whereby genetic ZW individuals develop into phenotypic males, designated as pseudomales [3].

Apoptosis, also known as programmed cell death, is characterized by unique morphological features and energy-dependent biochemical mechanisms [4]. It selectively eliminates redundant, damaged, or potentially harmful cells in the body [5,6], playing a crucial role in processes such as embryonic development, cell turnover, and immune response. Notably, *caspases* are key mediators of apoptosis. These enzymes contain a cysteine in their active site and are classified into initiator and effector *caspases*. Initiator *caspases* comprise *caspase2*, *caspase8*, *caspase9*, and *caspase10*, whereas effector *caspases* include *caspase3*, *caspase6*, *and caspase7*. After receiving apoptotic signals, initiator *caspases* are cleaved and activate effector *caspases* [7,8], which specifically cleave target proteins at aspartic acid residues, thereby inducing apoptosis [9,10,11]. *caspase3* interacts with *caspase8* and *caspase9* and plays a crucial role in apoptosis by degrading nuclear polymerase. It serves as a key executor of apoptosis. Evidence indicates that certain characteristic hallmarks of apoptosis, such as chromosome condensation and DNA fragmentation, are directly associated with *caspase3* [12]. In the *caspase*-mediated apoptotic signaling pathway, the convergence of the mitochondrial pathway and the death receptor pathway also occurs at *caspase3*. Studies have demonstrated that a low level of *caspase3* is present in the cytoplasm of normal spermatogonia and spermatocytes [13]. *caspase6* is an independent enzyme activated by *caspase3/caspase7* [14]. According to published data, the activity of *caspase6* is inhibited by some kinases. *caspase6* can also undergo self-processing without the action of other *caspase* family members [15]. *caspase6* is processed by *caspase7*, *caspase8* and *caspase10* and is also activated by *caspase1*. *caspase6* can also function as a non-canonical executioner or even as an inflammatory *caspase* [13].

*caspases* play a role in sex reversal and gonadal development. For example, during post-natal testis development and spermatogenesis in mice, the expression of *caspase3* is related to apoptosis. After sexual maturity, *caspase3* in spermatogonia can induce their apoptosis [16]. In fish, *caspase3* is expressed in the gonads of *Monopterus albus* at all developmental stages, especially with the highest expression level in the gonads during the vitellogenesis stage. The expression level shows a downward trend as the gonads develop towards the male direction [17], indicating its important role in the sex reversal process. In our previous studies, we found that high temperature can induce the sex reversal of genetic female Chinese tongue sole into a phenotypic male (pseudomale) [18,19], and the apoptotic signal in the gonads of pseudomale fish is stronger than that in males. A recent single-cell study in Chinese tongue sole testes revealed the reduced number of differentiated spermatogonia and pre-Lep cells in pseudomales compared to males. Considering the upregulated expression of the apoptosis-related gene *znrf4*, the following questions emerge [20].

Do *caspases* respond to high-temperature signals? How do they participate in sex differentiation or sex reversal?

A total of 10 caspase gene sequences were retrieved from the NCBI database, and six of these sequences were successfully obtained via gene cloning (*caspase1-like*, *caspase3a*, *caspase6*, *caspase8*, *caspase8-like*, *caspase9*). We analyzed their sequence characteristics, expression profiles, cellular localization, and in vitro siRNA knockdown in Chinese tongue sole. These data indicate that these genes play a role in the process of sex differentiation. Clarifying these issues will help us deeply understand the sex differentiation mechanism and is of great significance for the development of the Chinese tongue sole industry.

## 2. Results

### 2.1. Systematic Phylogenetic Tree Analysis of Caspase Genes

**Figure 1:**
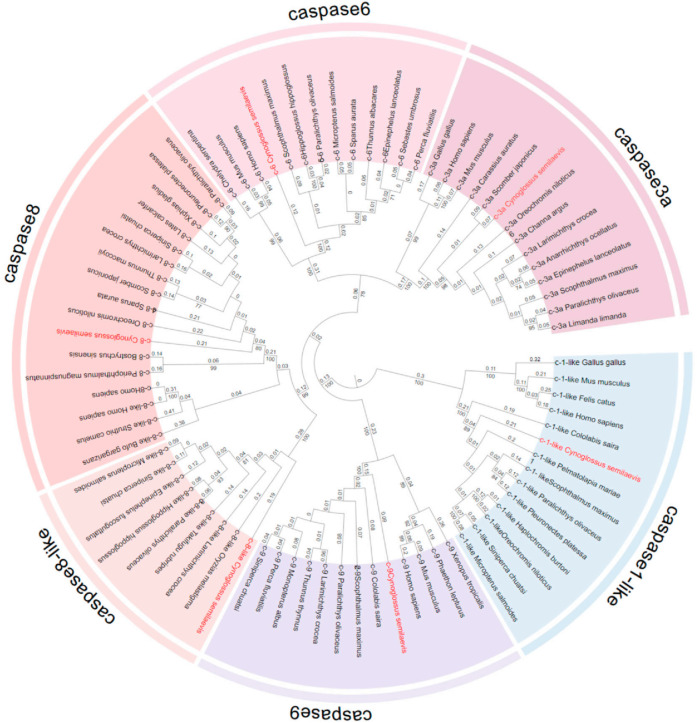
The sequences of *caspase1-like*, *caspase3a*, *caspase6*, *caspase8*, *caspase8-like*, and *caspase9* from multiple species form a phylogenetic tree. Chinese tongue sole is highlighted in red to indicate its evolutionary position. The numbers on the branches indicate the majority support values obtained from 1000 repeated simulations.

To investigate the evolutionary relationships among members of the *caspase* family, a phylogenetic tree was constructed. Phylogenetic tree analysis revealed that the *caspase* proteins of Chinese tongue sole cluster into two major clades: one consists of *caspase1-like*, and the other comprises *caspase6*, *caspase3a*, *caspase8*, *caspase9*, and *caspase8-like*. The latter clade is further subdivided into three subclades: *caspase9*, a subclade containing *caspase8* and *caspase8-like*, and another encompassing *caspase3a* and *caspase6* (Figure 1).

### 2.2. Sequence Analysis

The full-length coding sequences (CDSs) of six *caspase* genes were identified in Chinese tongue sole. The *caspase1-like* CDS spans 1170 bp and encodes 389 amino acids (aa), with a predicted molecular weight (MW) of 44.11 kDa and an isoelectric point (pI) of 5.77. The *caspase3a* CDS (843 bp) encodes a 280 aa protein (MW: 31.05 kDa; pI: 5.55), while the *caspase6* CDS (903 bp) comprises 300 aa (MW: 33.87 kDa; pI: 6.02). For the *caspase8* subfamily, the *caspase8* CDS spans 1467 bp and encodes 488 aa (MW: 55.81 kDa; pI: 5.26), whereas the *caspase8-like* CDS consists of 1161 bp and encodes 386 aa (MW: 43.61 kDa; pI: 6.95). Finally, the *caspase9* CDS spans 1338 bp and encodes a 445 aa polypeptide with a predicted MW of 50.07 kDa and a pI of 6.43.

**Figure 2:**
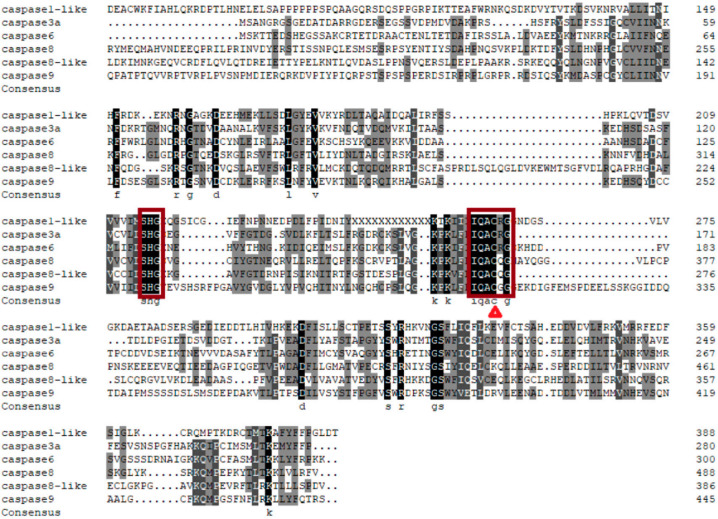
Amino acid sequence alignment. Red boxes indicate *caspase* active sites; red triangles denote highly conserved cysteine residues in *caspases*.

To further understand the conserved structures of the *caspase* gene family in Chinese tongue sole, a multiple sequence alignment analysis of *caspase* amino acid sequences revealed that all *caspase* sequences contain a conserved cysteine residue and active site at the same position (Figure 2).

### 2.3. Analysis of Expression Patterns in Different Tissues

**Figure 3:**
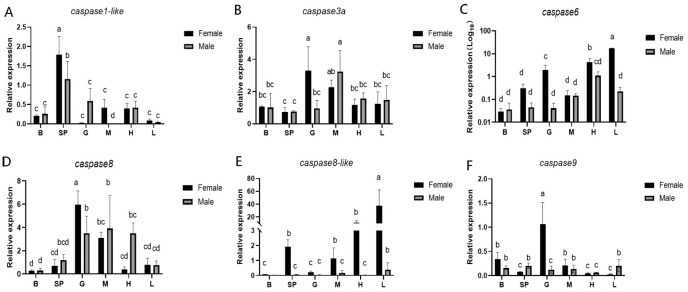
The expression levels of *caspase* in the different tissues of the Chinese tongue sole. (**A**) Expression of *caspase1-like* in various tissues. (**B**) Expression of *caspase3a* in various tissues. (**C**) Expression of *caspase6* in various tissues. (**D**) Expression of *caspase8* in various tissues. (**E**) Expression of *caspase8-like* in various tissues. (**F**) Expression of *caspase9* in various tissues. In multiple comparisons, different letters (a,b,c,d) indicate significant differences. The data were analyzed with SPSS 25.0 (IBM Corp., Armonk, NY, USA) using a one-way ANOVA and multiple comparisons by the Wohler and Duncan methods, and a *p*-value < 0.05 was considered the threshold for statistical significance. (**C**) is plotted on a log10 scale.

To explore the expression patterns of the *caspase* gene family in different tissues of Chinese tongue sole, the expression levels of these genes in six distinct tissues of the species were determined using q-PCR. qPCR analysis revealed that *caspase1-like*, *caspase3a*, *caspase6*, *caspase8*, *caspase8-like*, and *caspase9* are ubiquitously expressed in the brain, spleen, gonads, muscle, heart, and liver of male and female Chinese tongue sole. Tissue-specific expression patterns were observed: *caspase1-like* exhibited the highest expression in the spleen; *caspase3a* was highly expressed in the gonads and muscle; *caspase6* showed elevated expression levels in the gonads, muscle, and liver; *caspase8* had the highest expression level in the gonads; *caspase9* was the most abundantly expressed in the gonads. Notably, the *caspase8-like* expression level was significantly higher in all tissues of female fish compared to male counterparts (Figure 3).

### 2.4. Analysis of Caspase Expression Patterns in Female, Male, and Pseudomale Gonads

**Figure 4:**
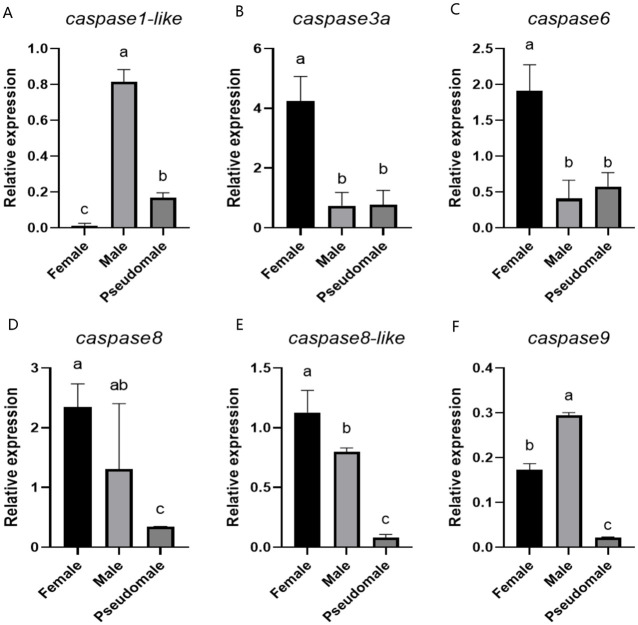
The expression levels of *caspase* in female, male, and pseudomale gonads of Chinese tongue sole. (**A**) Expression of *caspase1-like* in Chinese tongue sole. (**B**) Expression of *caspase3a* in Chinese tongue sole. (**C**) Expression of *caspase6* in Chinese tongue sole. (**D**) Expression of *caspase8* in Chinese tongue sole. (**E**) Expression of *caspase8-like* inChinese tongue sole. (**F**) Expression of *caspase9* in Chinese tongue sole. In multiple comparisons, different letters indicate significant differences. The data were analyzed with SPSS 25.0 (IBM Corp., Armonk, NY, USA) using a one-way ANOVA and multiple comparisons by the Wohler and Duncan methods, and a *p*-value < 0.05 was considered the threshold for statistical significance.

To investigate the expression patterns of the *caspase* gene family in the gonads of females, males, and pseudomale fish of Chinese tongue sole, qPCR was performed to determine their expression levels. qPCR analysis revealed that the expression levels of *caspase1-like* and *caspase9* were significantly higher in males than in females and pseudomales of Chinese tongue sole, whereas *caspase3a*, *caspase6*, *caspase8*, and *caspase8-like* exhibited significantly higher expression levels in females compared to males and pseudomales (Figure 4).

### 2.5. Analysis of Expression Patterns at Different Developmental Stages

**Figure 5:**
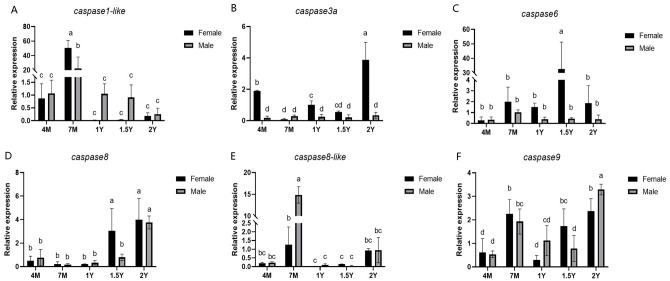
The expression levels of *caspase* in the gonads of Chinese tongue sole at different developmental stages. (**A**) *caspase1-like* at different gonadal developmental stages. (**B**) *caspase3a* at different gonadal developmental stages. (**C**) *caspase6* at different gonadal developmental stages. (**D**) *caspase8* at different gonadal developmental stages. (**E**) *caspase8-like* at different gonadal developmental stages. (**F**) *caspase9* at different gonadal developmental stages. In multiple comparisons, different letters (a,b,c,d) indicate significant differences. The data were analyzed with SPSS 25.0 (IBM Corp., Armonk, NY, USA) using a one-way ANOVA and multiple comparisons by the Wohler and Duncan methods, and a *p*-value < 0.05 was considered the threshold for statistical significance.

The expression patterns of *caspases* in male and female Chinese tongue sole at five developmental stages were further detected. The qPCR results indicate that *caspase3a*, *caspase6*, *caspase8*, *caspase9*, *caspase8-like*, and *caspase1-like* are expressed throughout all developmental stages. The expression levels of the *caspase3a*, *caspase8*, and *caspase9* genes peaked in both males and females at 2 yph. The *caspase6* gene reached its peak expression in females at 1.5 yph and in males at 2 yph. The *caspase1-like* and *caspase8-like* genes exhibited peak expression in both males and females at 7 mph (Figure 5).

### 2.6. In Situ Hybridization Results

**Figure 6:**
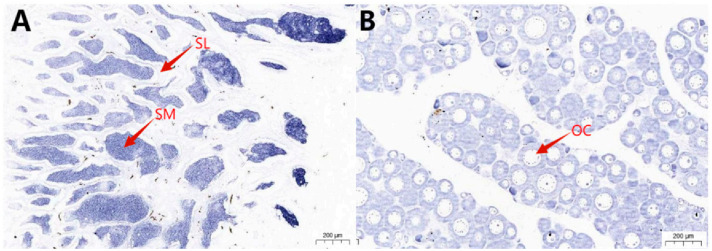

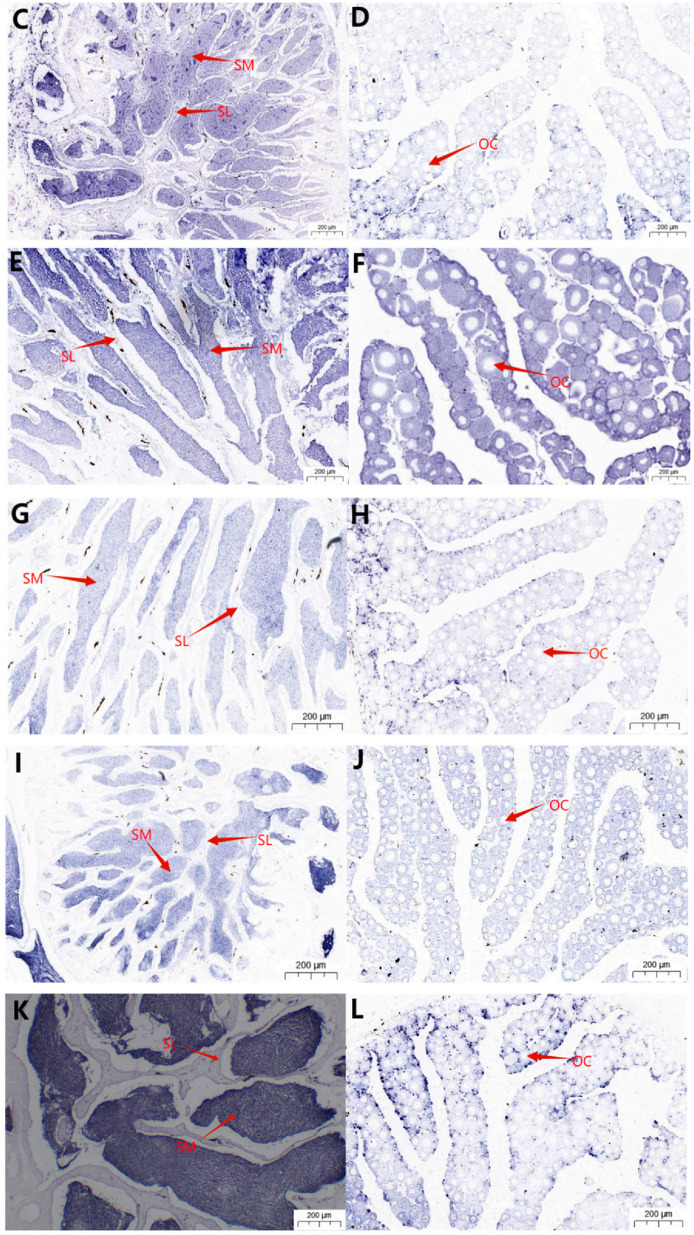
In situ hybridization results for T7 probes of *caspase* in Chinese tongue sole. (**A**,**B**): *caspase1-like*; (**C**,**D**): *caspase3a*; (**E**,**F**): *caspase6*; (**G**,**H**): *caspase8*; (**I**,**J**): *caspase8-like*; (**K**,**L**): *caspase9*. OC: Oocyte; SM: Sperm; SL: Sertoli cell. (See Figure A1 for in situ hybridization results for SP6 probes, negative control.)

To investigate the mRNA localization patterns of *caspase* genes in the gonadal tissues of Chinese tongue sole, ISH was performed on the gonadal tissues of 1-year-old Chinese tongue sole. The results demonstrated that the *caspase3a*, *caspase6*, *caspase8*, *caspase9*, *caspase8-like*, and *caspase1-like* genes are predominantly expressed in spermatocytes and oocytes (Figure 6).

### 2.7. Promoter Activity Analysis

**Figure 7:**
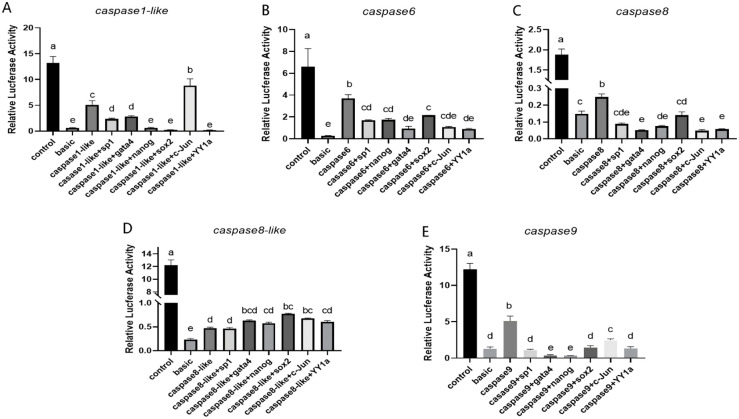
An assay of transcription factor binding site activity on *caspase* promoters. (**A**) Measurement of the Activity of Transcription Factor Binding Sites on the *caspase1-like* Promoter. (**B**) Measurement of the Activity of Transcription Factor Binding Sites on the *caspase6* Promoter. (**C**) Measurement of the Activity of Transcription Factor Binding Sites on the *caspase8* Promoter. (**D**) Measurement of the Activity of Transcription Factor Binding Sites on the *caspase8-like* Promoter. (**E**) Measurement of the Activity of Transcription Factor Binding Sites on the *caspase9* Promoter. In multiple comparisons, different letters (a,b,c,d,e) indicate statistically significant differences. The data were analyzed with SPSS 25.0 (IBM Corp., Armonk, NY, USA) using a one-way ANOVA and multiple comparisons by the Wohler and Duncan methods, and a *p*-value < 0.05 was considered the threshold for statistical significance.

**Figure A2:**
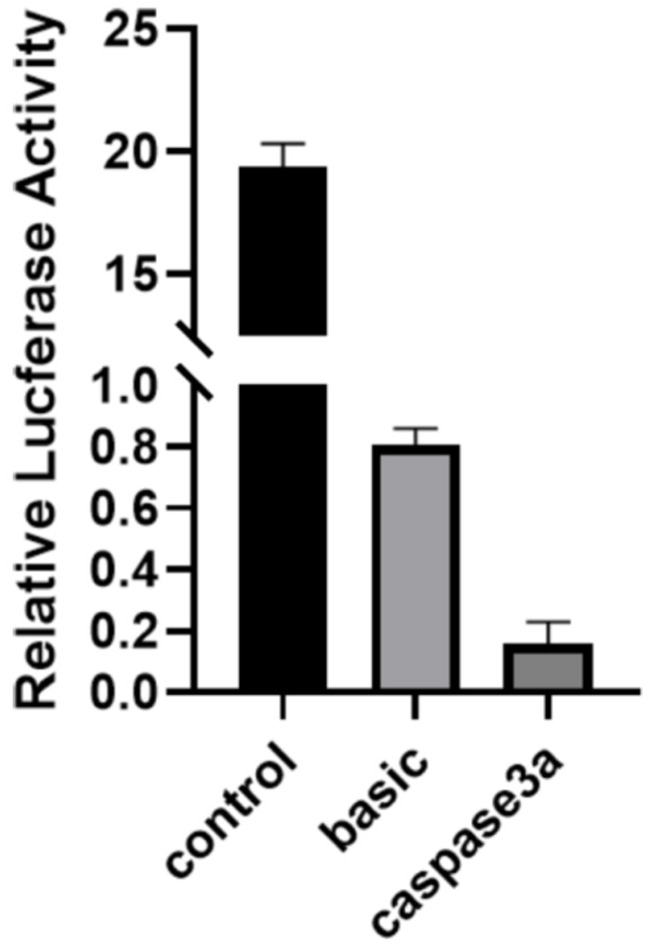
*caspase3a* promoter activity.

Based on the dual-luciferase reporter assay results, the firefly/Renilla luciferase activity ratio was significantly higher in the test groups than in the pGL3-basic transfection group (negative control). This indicates that the promoters of *caspase6*, *caspase8*, *caspase9*, *caspase8-like*, and *caspase1-like* are localized within the 2000–3000 bp upstream region of their respective coding sequences and exhibit strong promoter activity. The promoter regions of *caspase6*, *caspase8*, and *caspase9* exhibit reduced activity upon binding to transcription factors *sp1*, *gata4*, *nanog*, *sox2*, *yy1a*, and *c-Jun*. The *caspase1-like* promoter region shows decreased activity when combined with transcription factors *sp1*, *gata4*, *nanog*, *sox2*, and *yy1a*, and binding reduces promoter activity, while *c-Jun* binding increases it. The *caspase8-like* promoter region exhibits increased activity upon binding to transcription factors *sox2* and *c-Jun* (Figure 7). The *caspase3* promoter shows no detectable activity (Figure A2).

### 2.8. High-Temperature Response Experiment

**Figure 8:**
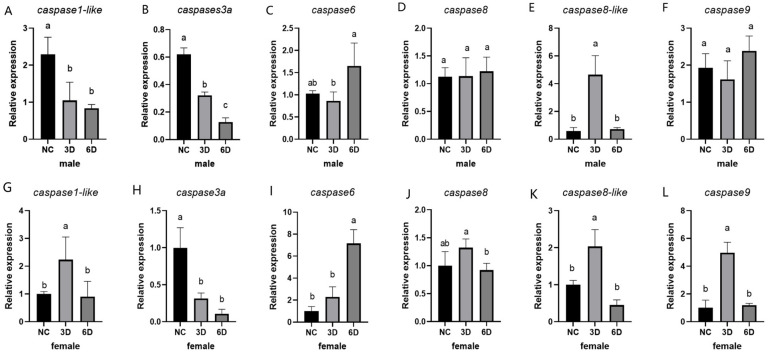
Expression of *caspase* in Chinese tongue sole after high-temperature treatment. (**A**–**F**) Expression of *caspase* in the gonads of male fish after high-temperature treatment. (**G**–**L**) Expression of *caspase* in the gonads of female fish after high-temperature treatment. In multiple comparisons, different letters (a,b,c) indicate significant differences. Data were analyzed with SPSS 25.0 (IBM Corp., Armonk, NY, USA) using one-way ANOVA and multiple comparisons by Wohler and Duncan methods, and *p*-value < 0.05 was considered threshold for statistical significance.

To investigate the mechanism by which *caspases* respond to temperature, we subjected female and male fish to high-temperature treatments. After high-temperature stimulation, significant changes occurred in the expression levels of *caspases* in both female and male fish. For *caspase1-like*, the expression level was lower in male fish compared to controls on both days 3 and 6 of high-temperature treatments. Female fish showed elevated expression levels on day 3 of high-temperature treatments, with no significant difference observed at 6 days. For *caspase3a*, the expression level was lower than that of controls in both sexes on both days 3 and 6 of high-temperature treatments. For *caspase6*, the expression level showed no significant difference in males, but it increased in females after 6 days; *caspase8* expression showed no significant difference in either sex after high-temperature treatment. For *caspase8-like*, the expression level increased in both sexes after 3 days of high-temperature treatment, with no significant difference observed after 6 days. For *caspase9*, the expression level increased in females after 3 days of high-temperature treatment, while no significant difference was observed in males after either 3 or 6 days of treatment (Figure 8).

### 2.9. Effects of Knockdown on Other Genes Following In Vitro Knockdown

**Figure 9:**
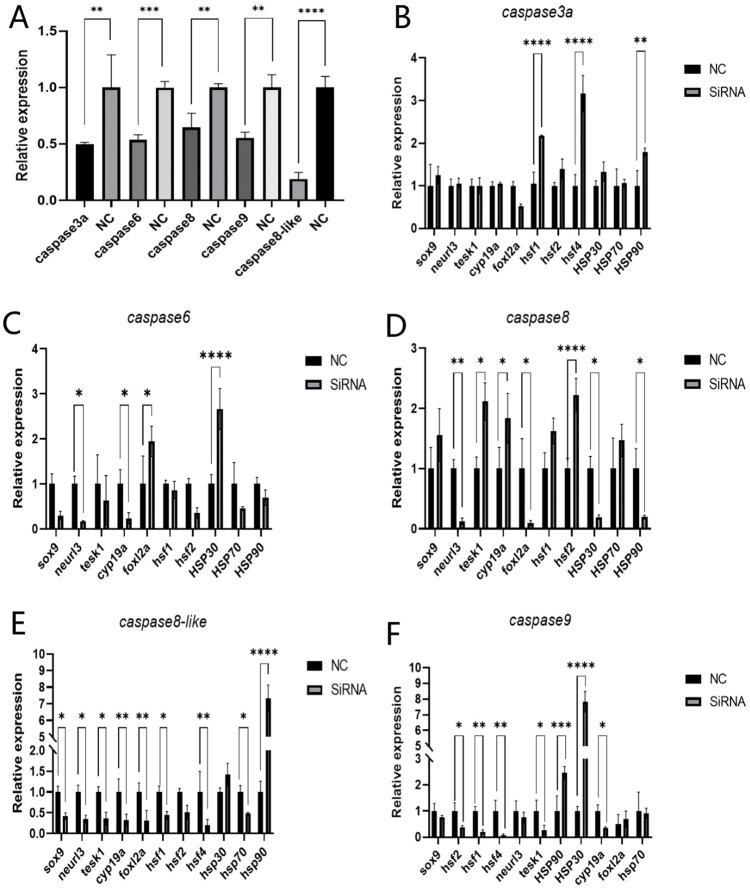
siRNA-mediated knockdown of *caspase* in Chinese tongue sole altered expression of other genes in testicular cells. (**A**): Interference efficiency of *caspase* siRNA. (**B**–**F**) Gene expression patterns in testicular cells after transfection with *caspase* siRNANC: negative control siRNA. Data were analyzed with SPSS 25.0 (IBM Corp., Armonk, NY, USA) using *t*-test. Data of each related gene were compared with NC, and *p*-value < 0.05 was considered threshold for statistical significance and indicated by *. (*: *p* ≤ 0.05, **: *p* ≤ 0.005, ***: *p* ≤ 0.0005, ****: *p* ≤ 0.0001.)

**Figure 10:**
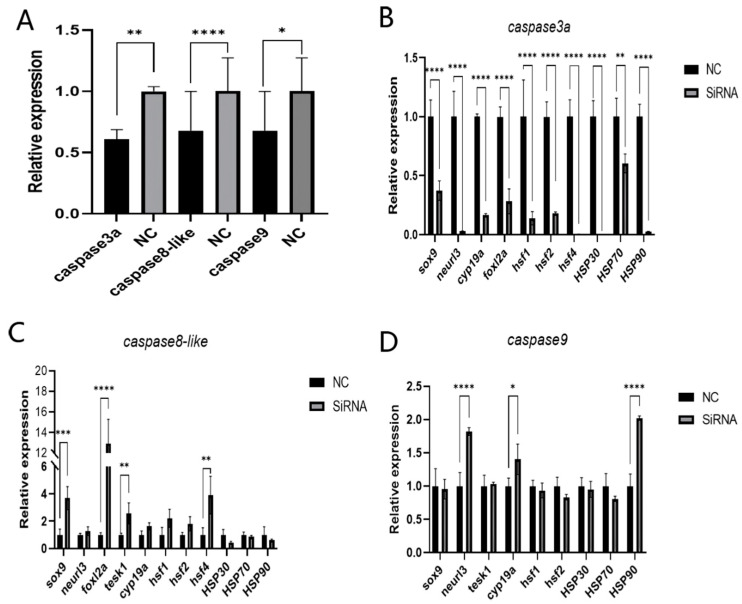
siRNA-mediated knockdown of *caspase* in Chinese tongue sole affects expression of other genes in ovarian cells. (**A**): Interference efficiency of *caspase* siRNA. (**B**–**D**) Gene expression patterns in ovarian cells after transfection with *caspase* siRNANC: negative control siRNA. Data were analyzed with SPSS 25.0 (IBM Corp., Armonk, NY, USA) using *t*-test. Data of each related gene were compared with NC, and *p*-value < 0.05 was considered threshold for statistical significance and indicated by *. (*: *p* ≤ 0.05, **: *p* ≤ 0.005, ***: *p* ≤ 0.0005, ****: *p* ≤ 0.0001.)

**Figure A3:**
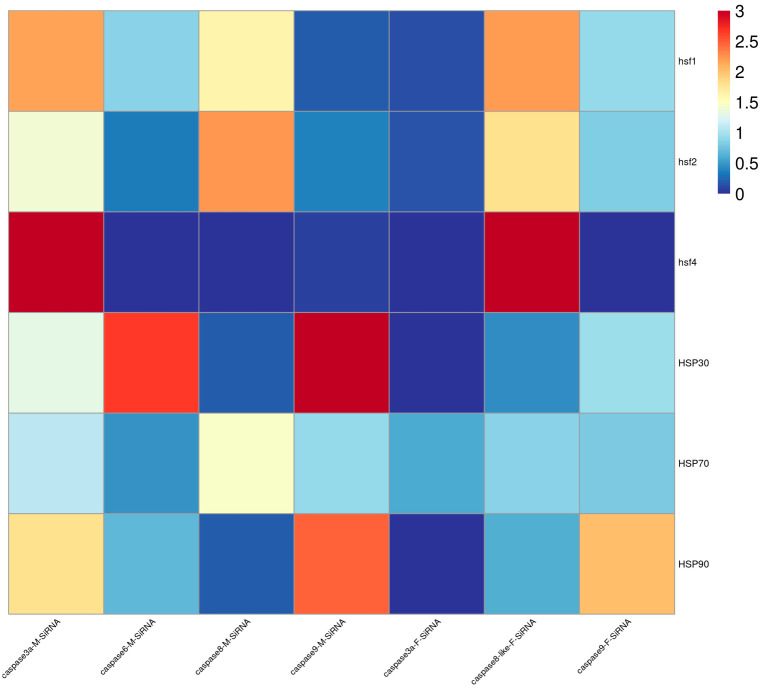
Heatmap of *hsf* and *hsp* genes.

**Figure A4:**
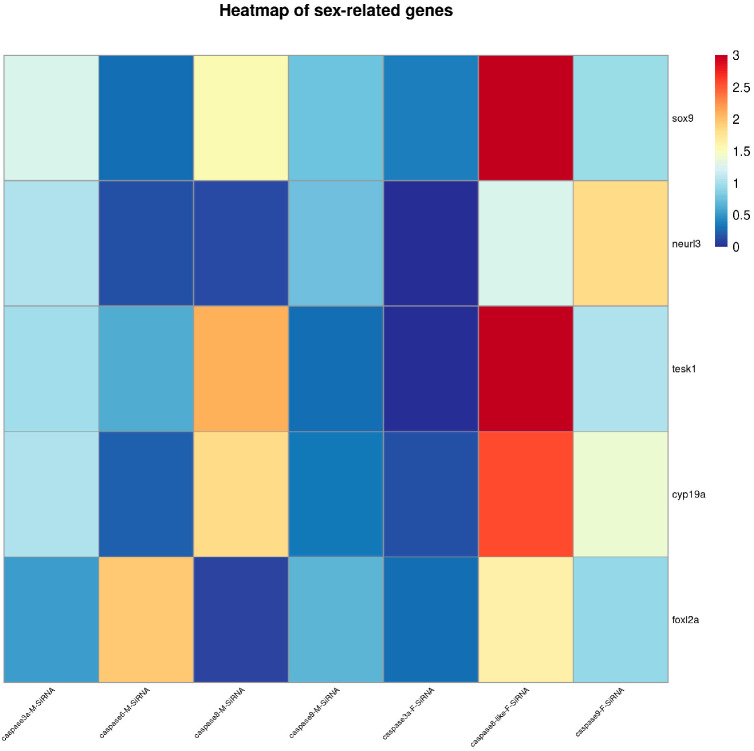
Heatmap of sex-related genes.

To analyze the regulatory effect of *caspase*, in vitro knockdown experiments were conducted. Following siRNA transfection into testicular cells, qRT-PCR analysis confirmed that, compared with the negative control (NC) group, *caspase3a*, *caspase6*, *caspase8*, *caspase8-like*, and *caspase9* exhibited robust interference effects. The results showed that *caspase3a* knockdown significantly increased the expression levels of *hsf1*, *hsf4*, and *hsp90*; *caspase6* knockdown significantly decreased the expression of *neurl3*, *cyp19a*, and *foxl2a* while significantly increasing *hsp30* expression; *caspase8* knockdown significantly reduced the expression of *neurl3*, *foxl2a*, *hsp30*, and *hsp90* but significantly increased the expression of *tesk1*, *cyp19a*, and *hsf2*; *caspase8-like* knockdown significantly decreased the expression of *sox9*, *neurl3*, *tesk1*, *cyp19a*, *foxl2a*, *hsf1*, *hsf4*, and *hsp70* while significantly increasing *hsp90* expression; and *caspase9* knockdown significantly reduced the expression of *tesk1*, *cyp19a*, *hsf1*, *hsf2*, and *hsf4* while significantly increasing *hsp30* and *hsp90* expression (Figure 9). Following siRNA transfection into ovarian cells, qRT-PCR analysis confirmed that, compared with the negative control (NC) group, the robust silencing of *caspase3a* and *caspase8-like* was validated. *caspase3a* knockdown resulted in a significant downregulation of *sox9*, *neurl3*, *cyp19a*, *foxl2a*, *hsf*, and *hsp* expression. *caspase8-like* was knocked down, and the expression levels of *sox9*, *foxl2a*, *tesk1*, and *hsf4* increased. After *caspase9* knockdown, the expression levels of *neurl3*, *cyp19a*, and *hsp90* increased (Figure 10). Overall, the RNA knockdown of *caspase8* in testicular cells and *caspase8-like* in ovarian cells led to the upregulation of most sex-related genes. Conversely, *caspase3a* (in both gonadal cells) and *caspase9* (in testicular cells) knockdown resulted in the downregulation of most sex-related genes. For *hsf*, *caspase3a/caspase8* knockdown in testicular cells and *caspase8-like* knockdown in ovarian cells upregulated most *hsf* genes, while *caspase6/caspase9* (testicular) and *caspase3a/caspase9* (ovarian) knockdown downregulated them. For *hsp*, *caspase3a/caspase8-like* knockdown in ovarian cells led to *hsp* gene downregulation (Figure A3 and Figure A4).

## 3. Discussion

*caspases* have been extensively studied in the context of immunity [21], whereas research on their roles in gonads remains limited. Thus, this study initiated an investigation into the functions of *caspases* in the gonads of Chinese tongue sole. Previous research indicates that apoptosis occurs during mammalian germ cell development, a process closely linked to the expression regulation of *caspase* family proteins. For instance, during the gonadal transformation from female to male in the three-spot wrasse (*Halichoeres trimaculatus*), oocyte apoptosis is tightly associated with *caspase3* expression [22]. Under thermal stress, oocyte apoptosis in the sand smelt (*Atherina presbyter*) correlates with robust *caspase3* expression [23]. With regard to Chinese tongue sole, our studies on *caspase* genes reveal their constitutive expression across all gonadal developmental stages. In situ hybridization further confirmed that *caspase* family genes are primarily localized in spermatocytes and oocytes. Phylogenetic tree analysis showed that Chinese tongue sole *caspase* genes cluster closely with homologous genes from other teleost species, reflecting the evolutionary conservation of the *caspase* gene family among bony fish.

As a key transcription factor in gonadal development, *gata4* typically modulates estrogen production during fish sex differentiation by regulating steroidogenic enzyme genes (e.g., *cyp19a*) [24]. *c-Jun*, a core member of the AP-1 transcription factor family, binds to AP-1 motifs in the promoters of target genes to activate the expression of apoptosis-related genes [25,26]. During the sexual differentiation of mouse external genitalia, *sp1* functions as a co-activator of the androgen receptor, regulating the expression of androgen-dependent male-specific genes [27]. Transcription factors *sp1*, *gata4*, *nanog*, *sox2*, and *yy1a* exert inhibitory effects on the expression of the *caspase6*, *caspase8*, *caspase9*, and *caspase8-like* genes. In contrast, c-Jun exerts an activating effect on the caspase1-like and caspase8-like genes. Collectively, these findings indicate that apoptosis-related *caspase* genes may participate in the sex differentiation process of Chinese tongue sole.

Temperature is the primary factor inducing sex reversal in Chinese tongue sole. Experimental data demonstrate that high temperature significantly modulates the expression of *caspase* genes, with females exhibiting a more robust response than males. Related studies indicate that, under heat stress, the expression of *caspase3* and *caspase9* in the gills of medaka (*Oryzias latipes*) increased significantly with increasing temperature, while *caspase1* expression was significantly downregulated [28]. Collectively, these findings indicate that heat stress likely exerts a significant regulatory effect on the expression of apoptosis-related genes in Chinese tongue sole. Through *caspase* gene knockdown experiments, we observed alterations in the expression of a series of heat shock transcription factors (*hsf*) and heat shock proteins (*hsp*). These findings suggest that *caspase* genes may interact with HSPs and HSFs to mediate the effects of high-temperature stress on sexual differentiation in Chinese tongue sole. *hsf* and *hsp* are core components of the organismal stress response to stimuli such as high temperature. Specifically, *hsf* act as key regulators of thermal stress responses, activating HSP expression to maintain cellular homeostasis and other physiological functions [29,30]. HSP90 stabilizes the anti-apoptotic protein Bcl-2, inhibits cytochrome c release from mitochondria, and facilitates Apaf-1-mediated apoptosome assembly, thereby suppressing the activation of *caspase3* and *caspase9* [31]. *hsp70* effectively blocks apoptosis through the dual inhibition of *caspase3* activity: directly binding to *caspase3* to prevent its activation and indirectly suppressing apoptosome formation [32,33].

Following siRNA interference in gonadal cell lines, the expression of multiple sex differentiation genes was affected. *foxl2a* and *cyp19a* are key genes for female differentiation in Chinese tongue sole. *foxl2a* activates the transcription of *cyp19a,* thereby facilitating estrogen biosynthesis. Estrogen serves as a critical signal for ovarian differentiation and maintenance, driving the differentiation of germ cells into oocytes [34,35]. Studies reveal that *foxl2a* positively regulates ovarian differentiation in mice. Sustained *foxl2* expression suppresses the abnormal differentiation of ovarian cells into testicular cells during mouse development, thereby maintaining their apparent female phenotype. Conversely, *foxl2a* deficiency leads to ovarian hypoplasia and even female infertility [36,37]. Following siRNA interference in ovarian cell lines of Chinese tongue sole, *caspase3a* knockdown resulted in a significant downregulation of *foxl2a* and *cyp19a* expression, whereas *caspase8-like* silencing led to a marked upregulation of *foxl2a* expression, and *caspase9* knockdown induced an increase in *cyp19a* expression. In testicular cell lines, *caspase* knockdown similarly elicited divergent changes in *foxl2a* and *cyp19a* expression. These observations suggest that *caspase* genes may be closely implicated in ovarian development. *neurl3* plays a critical role in male gonadal differentiation and spermatogenesis [38]; its expression was downregulated following *caspase6*, *caspase8*, and *caspase8-like* knockdown in testicular cell lines, indicating that *caspase* genes may have a positive regulatory role in spermatogenesis. *caspase8-like* exhibited sexually dimorphic expression, with significantly higher levels in females than in males. After heat stimulation, its expression is significantly upregulated in both male and female fish, which is worthy of attention.

The function of *caspase* is widely studied in inflammation and cell death. In this study, the function of *caspase* in sex differentiation was investigated. Its response to high temperature and close relation to *hsp* and *hsf* suggest its potential role in sex reversal, which provides a new perspective in *caspase* functionality. However, in vivo experiments such as gene editing or in vivo RNAi are required to check the phenotype and dissect the role of specific *caspase* genes.

## 4. Materials and Methods

### 4.1. Ethics Statement

In this study, the fish were anesthetized with MS-222 to alleviate pain. The animal experiment was inspected and approved by the Institutional Animal Care and Use Committee at the Yellow Sea Fisheries Research Institute, CAFS (Approval No.:YSFRI-2022035; Date: 2 July 2022).

### 4.2. Preparation of Samples

Chinese tongue sole were sourced from the High-tech Experimental Base in Haiyang City, Shandong Province. Gonadal tissue samples were collected from three 1.5-year-old (yph) pseudomale fish. Additionally, spleen, heart, liver, muscle, brain tissue, and gonadal tissue samples were collected from three male and three female fish. These samples were immediately frozen in liquid nitrogen and then stored in a −80 °C freezer. Gonadal samples were harvested from fish at different developmental stages: 4 and 7 months post-hatch (mph) and 1 yph, 1.5 yph, and 2 yph (three fish/time point). Each gonadal specimen was partitioned into two aliquots: one for RNA extraction (stored at −80 °C) and the other fixed in 4% paraformaldehyde (PFA) for in situ hybridization (ISH). Caudal fins were clipped and preserved in absolute ethanol for DNA extraction and genetic sex determination.

### 4.3. Primer Synthesis and Design

All primers were designed using Primer5, with β-actin [39] as the internal reference gene. The primer sequences are shown in Table 1. The primers were synthesized by Beijing Ruiboxingke Biotechnology Co., Ltd. (Beijing, China).

### 4.4. Genomic DNA Extraction and Genetic Identification

Genomic DNA was extracted from caudal fin clips using the Marine Animal DNA Kit (Tiangen, Beijing, China) according to the manufacturer’s protocol. PCR amplification was performed with 2 × Takara Mix (Takara, Kusatsu, Japan) using the sex-specific primers Sex-F and Sex-R (Table 1), which were validated by Liu et al. [40] for Chinese tongue sole. PCR products were resolved on a 2% agarose gel. Genotypic females exhibited two distinct bands, whereas genotypic males displayed a single band. Individuals characterized by a female genotype but a testicular phenotype were identified as pseudomales.

### 4.5. Total RNA Extraction and cDNA Synthesis

Total RNA was extracted from samples using Trizol reagent (Invitrogen, Carlsbad, CA, USA). RNA integrity, concentration, and quality were assessed using 1% agarose gel electrophoresis and a micro-spectrophotometer (DNA/protein analyzer P100+) (Thermo, Waltham, MA, USA). All procedures were conducted under RNase-free conditions at 4 °C to prevent RNA degradation. Complementary DNA (cDNA) was synthesized with the TaKaRa PrimeScript™ RT reagent Kit with gDNA Eraser (Perfect Real Time; Takara, Japan), following the manufacturer’s instructions. The reverse-transcribed products were stored at −20 °C.

### 4.6. Cloning of Caspase Genes

Based on the sequence information of *caspase1-like*, *caspase3a*, *caspase6*, *caspase8*, *caspase8-like,* and *caspase9* in Chinese tongue sole on NCBI (https://www.ncbi.nlm.nih.gov, Gene ID = 103378482/103383467/103383854/103396237/103396239/103385017, accessed on 6 October 2024), primers (Table 1) were designed to clone the CDS regions of *caspases*. The cDNA from Chinese tongue sole tissues was selected as the template, and the total amplification system was 50 μL. The polymerase chain reaction (PCR) program was as follows: 98 °C for 5 min, followed by 38 cycles of 98 °C for 10 s, 55 °C for 5 s, 68 °C for 20 s, and finally an extension at 72 °C for 5 min. Amplicons were resolved by agarose gel electrophoresis, and the target fragments were excised and recovered. The FastPure Gel DNA Extraction Mini Kit (Vazyme, Nanjing, China) was used for purification and recovery. The recovered products were ligated to the pEASY-T1 vector. A total of 4 μL of the product and 1 μL of pEASY-T1 were mixed, and ligation was carried out in a PCR instrument at 25 °C for 30 min. Subsequently, the ligated products were transformed into Escherichia coli. Positive clones were identified via colony PCR and confirmed through Sanger sequencing (Ruibo, Qingdao, China).

### 4.7. Sequence Analysis and Phylogenetic Tree Construction

The results of amino acid sequence alignment and assembly of the *caspase* family were processed on the ExPASy website (https://web.expasy.org/protparam/, accessed on 7 October 2024) to predict the coding sequences, molecular weights, and isoelectric points. The SMART (http://smart.embl-heidelberg.de/, accessed on 7 October 2024) was employed to identify conserved domains. DNAMAN9.0 software (https://www.dnaman.net/) was used for multiple sequence alignment. A phylogenetic tree was constructed via the Neighbor-Joining (NJ) method using MEGA 11.0, with topological reliability assessed through 1000 bootstrap replicates.

### 4.8. Real-Time Fluorescent Quantitative PCR

Specific primers (Table 1) were designed to determine the spatio-temporal expression profiles of *caspase* genes, including tissue distribution (gonad, liver, spleen, gill, brain, and kidney) and developmental expression patterns (4, 7 mph; 1, 1.5, and 2 yph), as well as expression in the gonads of females, males, and pseudomales at 1.5 yph. β-actin [39] served as the internal control for normalization. qPCR was performed on a 7500 Fast Real-Time PCR System (Applied Biosystems, Carlsbad, CA, USA) with 10-fold diluted reverse-transcribed cDNA as the template, and each group included 3 biological replicates. The 10 μL qPCR reaction mixture contained 2 μL of cDNA template, 5 μL of THUNDERBIRD^®^ Next SYBR^®^ qPCR Mix (TOYOBO, Osaka, Japan), 0.2 μL each of forward and reverse primers, and 2.6 μL of ddH_2_O. The qPCR program was as follows: pre-denaturation at 95 °C for 30 s; followed by 40 cycles of denaturation at 95 °C for 5 s and annealing/extension at 60 °C for 30 s; and a final melting curve step at 95 °C for 15 s. Post-amplification, melting curve analysis was conducted to verify the specificity of the amplicons. Relative mRNA expression levels were quantified using the 2^−ΔΔCt^ method. Data were analyzed via a one-way ANOVA followed by multiple comparison tests using SPSS. Statistical significance was set at *p* < 0.05.

### 4.9. In Situ Hybridization

Based on the coding sequence (CDS) regions of the *caspase* genes, probe primers were designed (Table 1) and synthesized by Beijing RiboBio Co., Ltd. (China). PCR amplification was performed in a 50 μL reaction volume using these primers and the high-fidelity enzyme 2 × TOROBlue^®^ Flash KOD DyeMix (TOROIVD, Shanghai, China). The reaction mixture contained 2 μL of diluted gonadal cDNA (template), 1.5 μL each of forward and reverse primers, 25 μL of 2 × TOROBlue^®^ Flash KOD DyeMix, and 20 μL of RNase-Free ddH_2_O. The PCR program was as follows: denaturation at 98 °C for 10 s, annealing at 60 °C for 5 s, and extension at 68 °C for 5 s (35 cycles), followed by a final extension at 72 °C for 7 min and storage at 4 °C. The PCR products were purified and recovered. In vitro transcription was conducted in a 20 μL total reaction volume with T7 or SP6 RNA polymerase, following the instructions of the Roche In Vitro Transcription Kit and the Digoxigenin (DIG) Probe Manual (Roche, Penzberg, Germany). This yielded DIG-labeled sense and antisense RNA probes. The probes were purified via the LiCl precipitation method and stored at −80 °C. Gonadal tissue sections were prepared from 1-year-old male and female Chinese tongue sole (section thickness 5 µm). Sections were deparaffinized in xylene, rehydrated through a graded ethanol series, and fixed. Prehybridization was performed at 60 °C for 4 h using the prepared prehybridization buffer. The probes were heat-denatured and diluted to a final concentration of 0.2 μg/mL in hybridization buffer. The sections were then incubated overnight at 60 °C with this probe-containing hybridization buffer. After hybridization, the sections were washed, blocked with goat serum-containing blocking solution at room temperature for 4 h, and subsequently incubated overnight with an anti-digoxigenin antibody. Colorimetric detection was performed using the BCIP/NBT Kit (Roche, Germany). Images were acquired using a Nikon ECLIPSE 80i microscope and a Pannoramic MIDI II digital slide scanner (3DHISTECH, Budapest, Hungary).

### 4.10. Caspase Promoter Activity Analysis

#### 4.10.1. Construction of Promoter Recombinant Vectors

Based on the genomic sequences of the *caspase* genes, primers containing HindIII restriction endonuclease recognition site linkers were designed using Primer5 software to amplify their promoter regions. Genomic DNA was used as the template for the PCR amplification of the upstream fragments of the *caspase* 5′UTR (Table 2). The PCR products were purified and recovered using a gel extraction kit and stored at −20 °C. The pGL3-basic vector was subjected to single-enzyme digestion with HindIII restriction endonuclease. Following digestion, the vector was purified, recovered, and verified by electrophoresis to confirm successful digestion. The purified promoter fragments were cloned into the linearized pGL3-basic vector using a seamless cloning kit (TOROIVD, Shanghai, China) to generate pGL3-*caspase* recombinant plasmids. These constructs were transformed into competent *Escherichia coli* cells, and positive clones were confirmed by sequencing (Qingke, Qingdao, China). After verifying sequence fidelity against NCBI references, endotoxin-free recombinant plasmids were isolated using a mid-scale plasmid extraction kit (Tiangen, Beijing, China) according to the manufacturer’s protocol.

#### 4.10.2. Cloning of Transcription Factors and Construction of Expression Vectors

Transcription factor binding sites (*c-Jun*, *sp1*, *gata4*, *nanog*, *sox2*, *yy1a*) in Chinese tongue sole *caspase* gene promoters were predicted using the AnimalTFDB4 online tool (https://guolab.wchscu.cn/AnimalTFDB4//#/, accessed on 2 January 2025.). TF-specific primers incorporating HindIII restriction sites were designed to amplify the full-length coding sequences (CDSs) of these factors. The PCR amplicons were purified and recovered. The pcDNA3.1 expression vector was linearized with HindIII and verified via agarose gel electrophoresis. Seamless cloning, ligation, and transformation were performed to construct recombinant vectors by inserting the transcription factor coding regions into the digested pcDNA3.1. The recombinant vectors were transformed into Escherichia coli competent cells and sent for sequencing. After confirming sequence accuracy, plasmids were extracted using a commercial kit and stored at −20 °C.

#### 4.10.3. Human Embryonic Kidney Cell (HEK293T) Culture

Human embryonic kidney (HEK) 293T cells were employed for cell-based assays in this study. The cells were maintained in high-glucose DMEM supplemented with 10% (*v*/*v*) fetal bovine serum (FBS; Gibco, Burlington, ON, Canada), without the addition of growth factors or antibiotics, at 37 °C in a humidified atmosphere containing 5% CO_2_. Upon reaching 80–90% confluence, the cells were passaged at a split ratio of 1:5. All cell culture procedures were performed under strict aseptic conditions within a pre-sterilized laminar flow hood to ensure a contamination-free environment.

#### 4.10.4. Promoter Activity Assay and Co-Transcription

Upon reaching 60–80% confluence, 293T cells were seeded into 24-well plates for transfection. Initially, cells were transfected with pGL3-caspase, pGL3-basic (negative control), or pGL3-Control to evaluate basal promoter activity, followed by co-transfection with transcription factor expression plasmids. Each treatment was performed in quadruplicate to ensure statistical reproducibility. The transfection complex (25 μL) consisted of 800 ng of reporter plasmid, 40 ng of pRL-TK internal control, and 1 μL of Lipofectamine 8000™ (Beyotime, Shanghai, China) in high-glucose DMEM.

At 48 h post-transfection, dual-luciferase activities were quantified. The culture medium was aspirated, and cells were incubated with 200 μL of lysis buffer for 5 min. Working solutions were prepared under light-shielded conditions. A 100 μL aliquot of cell lysate was transferred to an opaque 96-well plate, where firefly luciferase activity was first measured using a multimode microplate reader. Subsequently, 100 μL of Renilla luciferase assay solution was added to each well to determine Renilla luciferase luminescence. The relative promoter activity was expressed as the ratio of firefly to Renilla luciferase luminescence.

### 4.11. In Vivo High-Temperature Experiment

Fifty-day-old Chinese tongue sole juveniles obtained from the Weizhuo Experimental Base (Tangshan, China) were selected for high-temperature stress experiments. The juveniles were randomly assigned to three groups (n = 70 per group) under a 12 h light/12 h dark (12L:12D) photoperiod and a salinity of 25–30‰. The control group was maintained at 24 °C for six consecutive days. Experimental groups 1 and 2 were subjected to 30 °C for three and six days, respectively; for these groups, the water temperature was gradually elevated to 30 °C prior to the initiation of the experiment. Fish were fed every other day, and 100% of the rearing water was renewed daily. At the end of the trial, gonadal tissues were harvested from three males and three females per group, immediately snap-frozen in liquid nitrogen, and stored at −80 °C for subsequent molecular analysis (refer to Section 4.8 for detailed procedures).

### 4.12. Germ Cell Culture and siRNA Knockdown

Specific siRNAs targeting *caspase* genes and a non-targeting negative control (NC) siRNA were synthesized by Sangon Biotech (Shanghai, China). Primary testicular and ovarian cell lines (predominantly somatic cells) from Chinese tongue sole were established and maintained in our laboratory [41,42]. Cells were cultured in Leibovitz’s L-15 medium supplemented with 20% fetal bovine serum (FBS), 5 ng/mL bFGF (Invitrogen, Carlsbad, CA, USA), 5 ng/mL EGF, 27.5 μmol/L β-mercaptoethanol, and 5% antibiotics at 24 °C. Upon reaching 80–90% confluence, cells were passaged using trypsin digestion.

For RNAi experiments, cells were seeded into 12-well plates and transfected at 60–80% confluence using the CPRegent Transfection Kit (RiboBio, Guangzhou, China). Briefly, 3 μL of siRNA was incubated with 60 μL of 1× CP buffer and 5 μL of CPRegent for 5–10 min to form transfection complexes, which were then added to the wells. Each experimental group included three biological replicates. Total RNA was extracted 48 h post-transfection using TRIzol reagent (Ambion, Austin, TX, USA) and reverse-transcribed into cDNA. Knockdown efficiency was quantified by qPCR on an ABI 7500 Fast Real-Time PCR System using the 2^−ΔΔCt^ method. A downstream analysis of sex differentiation genes was performed only after significant silencing of the target *caspase* genes was confirmed.

## 5. Conclusions

This study employed qPCR, ISH, and siRNA interference to investigate the expression patterns and biological functions of *caspase* genes in male and female Chinese tongue sole. The results demonstrated that *caspase* genes are constitutively expressed throughout all gonadal developmental stages of Chinese tongue sole, with peak expression detected between 7 months and 2 years of age. ISH analysis localized *caspase* gene signals primarily to spermatocytes and oocytes. Under high-temperature stress, significant alterations in *caspase* gene expression were observed in the gonads. Following the siRNA-mediated knockdown of caspase genes in testis cell lines, the expression of multiple sex differentiation-related genes—including *sox9*, *neurl3*, *cyp19a*, *tesk1*, and *foxl2a*—was dysregulated. Notably, a suite of heat shock proteins (*hsp30*, *hso70*, *hsp90*) and heat shock transcription factors (*hsf1*, *hsf 2*, *hsf 4*) also exhibited significant expression changes. Collectively, these findings suggest that *caspase* genes may respond to high-temperature stimuli and play a critical role in the sex differentiation of Chinese tongue sole, providing a foundation for the further exploration of the molecular mechanisms underlying *caspase*-mediated regulation in this process.

## Figures and Tables

**Table 1:** Primer sequences.

Primer	Sequences (5′–3′)	Purpose
SexF	CCTAAATGATGGATGTAGATTCTGTC	Sex identification
SexR	GATCCAGAGAAAATAAACCCAGG	
*caspase1-like* F	TACAGCGGACATTGAGCA	PCR
*caspase1-like* R	GGTATTTAATAAGAAACCCACA	
*caspase3a* F	GCTTCAGCTCGTCACCTCT	
*caspase3a* R	AACAGCACCTCAGCCAAAT	
*caspase6* F	GGGCTTGTGGAAACTCAG	
*caspase6* R	GCTAAAATCACCTCGTAA	
*caspase8* F	GTGAAGATTGCGGAAGTAG	
*caspase8* R	AACTGTGCTGTTGGAAGAC	
*caspase8-like* F	CGCTGTTCTTACTGCGACTC	
*caspase8-like* R	TGCCACCCTGGAAATCTTA	
*caspase9* F	GCAAGACAATGCGAATGAAGAAAACTGT	
*caspase9* R	GTTTCGCCTGACCTGACCTAGCTTCGTG	
*caspase1-like* qF	GGGTGAGGCCCAACTTTGTA	qPCR
*caspase1-like* qR	TGAACTTCCAGCATGCCTCA	
*caspase3a* qF	TCACTTTTCCGGGGTGATCG	
*caspase3a* qR	CGCGTGTCATTATGTGCTGG	
*caspase6* qF	GCAGTACGGAGATTCCCTGG	
*caspase6* qR	ATCGAAGCAAAGCAAGGCAC	
*caspase8* qF	CAGCAAGACTGCCCTGGATT	
*caspase8* qR	CATAACTTGGCCGGGACTCA	
*caspase8-like* qF	TGTGTCAGCGTGGGGTTTTA	
*caspase8-like* qR	CACACGGCTGAGGATAGTCG	
*caspase9* qF	GCGAATGAAGAAAACTGTTATCGG	
*caspase9* qR	GTCTCTGACCAGTTCTCTGGC	
*β-actin* F	CCTTGGTATGGAGTCCTGTGGC	
*β-actin* R	TCCTTCTGCATCCTGTCGGC	
*caspase1-like* probeF	TGTAAGCAGGGCCTCCAAAG	ISH
*caspase1-like* probeR	TTTGATGGGTCTGCCTGGTG	
*caspase3a* probeF	ACAGGCATGAATCAGCGGAA	
*caspase3a* probeR	GGATCTTTGTGGTCCCGTCA	
*caspase6* probeF	AACAGGGACATGATTTCCTGG	
*caspase6* probeR	GCTTTGCTTCGATGCTCACC	
*caspase8* probeF	TGCCAAGGAAATGCCTACCA	
*caspase8* probeR	ACAGTCCCTTGCTGACGTTT	
*caspase8-like* probeF	GGCAGAAGTTTTCAGCTGGC	
*caspase8-like* probeR	TTTCGGTTTCCCACCGAGAG	
*caspase9* probeF	GCGAATGAAGAAAACTGTTATCGG	
*caspase9* probeR	GCTGGAGCTCCGTTCTGTAG	
*hsf1* qF	ATCGACTCCAGGATCAACGC	qPCR
*hsf1* qR	ATATTTGGGCACGGAGTGGG	
*hsf2* qF	GACTGAGAACGGAGCGATTT	
*hsf2* qR	GTTTGCCGGTTTGCATCATT	
*hsf4* qF	CTCCAGTCCTGCTTCAAAGT	
*hsf4* qR	GCCTTTGTCTCTGGTGTCA	
*hsp90* qF	GGAGCACGACCATGACAAGT	
*hsp90* qR	GATCCTCCCAGTCGTTGGTC	
*hsp70* qF	AGGGACTCTGGACGTGTCTT	
*hsp70* qR	CTGGTTTGGACGGTCAGGTT	
*hsp30* qF	ACTCTCAGCTCAGTCCACTC	
*hsp30* qR	ACAGACGTCTTCTTCTTCTTGTCC	
*foxl2a* qF	CCGGCCTGTGAAGAC	
*foxl2a* qR	TGCAGGTACTTAGGCG	
*sox9* qF	AAGAACCACACAGATCAAGACAGA	
*sox9* qR	TAGTCATACTGTGCTCTGGTGATG	
*cyp19a* qF	GGTGAGGATGTGACCCAGTGT	
*cyp19a* qR	ACGGGCTGAAATCGCAAG	
*neurl3* qF	CTGGTGTTTAGCAGCCGTCCT	
*neurl3* qR	CCAGAACTCCAGCACTGACCC	
*tesk1* qF	GCAGAAACTCTCTCACCCCAACA	
*tesk1* qR	CCAGACCAAAGTCCGTCACCA	

**Table 2:** Promoter primer sequences.

Primer	Sequences (5′–3′)	Purpose	Primer
*caspase1-like* pF	CTTTGACAGCGTGTAGCACTG	Promoter cloning	1952
*caspase1-like* pR	AGGGGAAAGCTAAGCTACAT		
*caspase3a* pF	GTCCTCCCAGTCTTTGTCATCCTG		1454
*caspase3a* pR	TGGAAGACTGTGTAGATCAGGGG		
*caspase6* pF	AGAAACAGGCAGCGCACTTA		3088
*caspase6* pR	ATTGGCTATCGTGCCTGTCA		
*caspase8* pF	TCCTCCCTCAGCTCTTTGCTG		1852
*caspase8* pR	TTACGTGCGTCAATATGGTGGT		
*caspase8-like* pF	TTATCGGACATCTCTAGTGCAAC		3895
*caspase8-like* pR	TTCCTTGGCTGACATTATGAA		
*caspase9* pF	TACCTACTTTGTAGAGAAGAGGA		3104
*caspase9* pR	TATTGTCTTGCACTCTCCATTTC		

**Figure A1:**
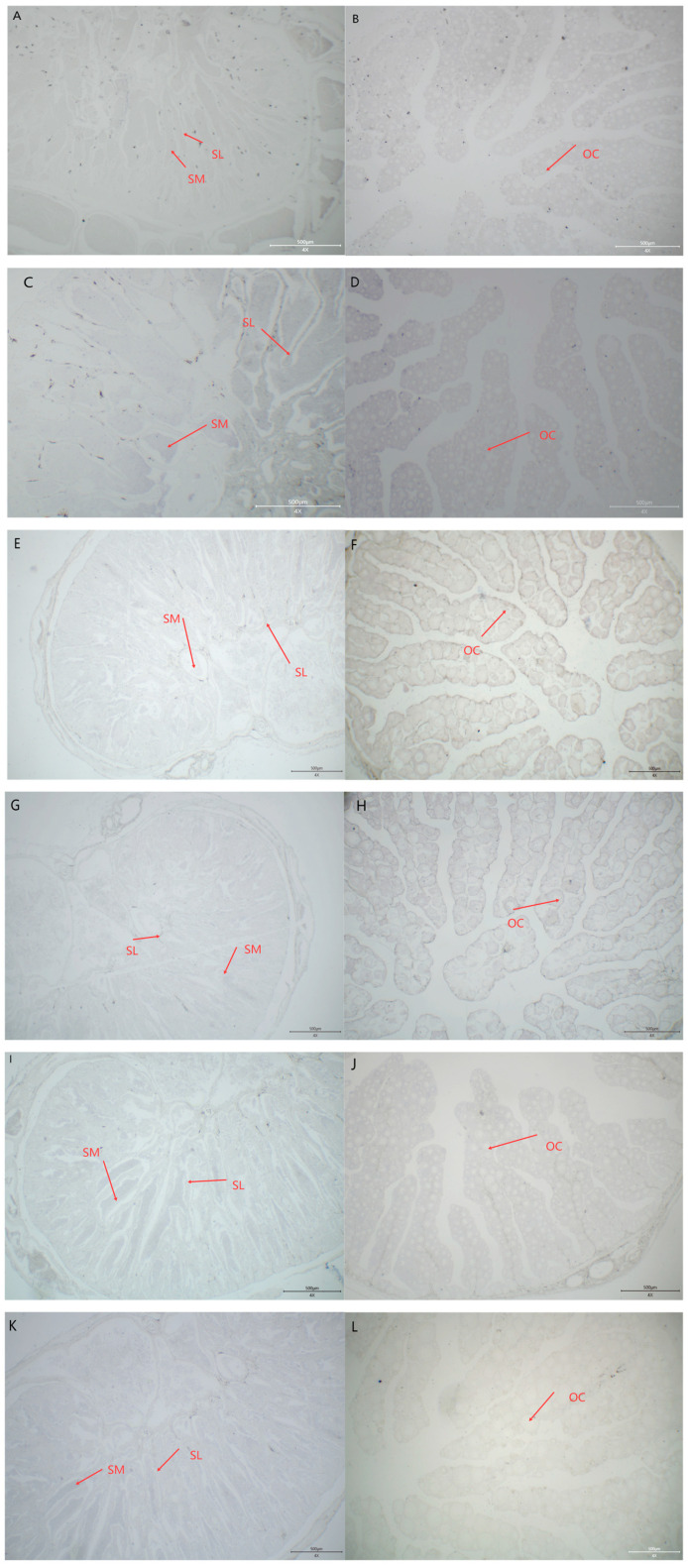
In situ hybridization results for SP6 probes of *caspase* in Chinese tongue sole. (**A**): *caspase1-like* sense probe in testis. (**B**): *caspase1-like* sense probe in ovary. (**C**): *caspase3a* sense probe in testis. (**D**): *caspase3* sense probe in ovary. (**E**): *caspase6* sense probe in testis. (**F**): *caspase6* sense probe in ovary. (**G**): *caspase8* sense probe in testis. (**H**): *caspase8* sense probe in ovary. (**I**): *caspase8-like* sense probe in testis. (**J**): *caspase8-like* sense probe in ovary. (**K**): *caspase9* sense probe in testis. (**L**): *caspase9* sense probe in ovary.

## Data Availability

The original contributions presented in this study are included in the article. Further inquiries can be directed to the corresponding author.

## Abbreviations

The following abbreviations are used in this manuscript:

B	Brain
SP	Spleen
G	Gonadal
M	Muscle
H	Heart
L	Liver
